# Automatic labeling of the fanning and curving shape of Meyer’s loop for epilepsy surgery: an atlas extracted from high-definition fiber tractography

**DOI:** 10.1186/s12883-019-1537-6

**Published:** 2019-11-28

**Authors:** Yong-Zhi Shan, Zhen-Ming Wang, Xiao-Tong Fan, Hua-Qiang Zhang, Lian-Kun Ren, Peng-Hu Wei, Guo-Guang Zhao

**Affiliations:** 1 Department of Neurosurgery, Xuanwu Hospital, Capital Medical University, No. 45 Changchun Street, Xuanwu District, Beijing, 100053 China; 2 Department of Radiology, Xuanwu Hospital, Capital Medical University, Beijing, 100053 China; 3 Department of Neurology, Xuanwu Hospital, Capital Medical University, Beijing, 100053 China

**Keywords:** Human connectome project, Medial temporal lobe epilepsy, Meyer’s loop, Anterior temporal lobe resection, Diffusion spectrum imaging

## Abstract

**Background:**

Visual field defects caused by injury to Meyer’s loop (ML) are common in patients undergoing anterior temporal lobectomy during epilepsy surgery. Evaluation of the anatomical shapes of the curving, fanning and sharp angles of ML to guide surgeries is important but still challenging for diffusion tensor imaging. We present an advanced diffusion data-based ML atlas and labeling protocol to reproduce anatomical features in individuals within a short time.

**Methods:**

Thirty Massachusetts General Hospital-Human Connectome Project (MGH-HCP) diffusion datasets (ultra-high magnetic gradient & 512 directions) were warped to standard space. The resulting fibers were projected together to create an atlas. The anatomical features and the tractography correspondence rates were evaluated in 30 MGH-HCP individuals and local diffusion spectrum imaging data (eight healthy subjects and six hippocampal sclerosis patients).

**Results:**

In the atlas, features of curves, sharp angles and fanning shapes were adequately reproduced. The distances from the anterior tip of the temporal lobe to the anterior ridge of Meyer’s loop were 23.1 mm and 26.41 mm on the left and right sides, respectively. The upper and lower divisions of the ML were revealed to be twisting. Eighty-eight labeled sides were achieved, and the correspondence rates were 87.44% ± 6.92, 80.81 ± 10.62 and 72.83% ± 14.03% for MGH-HCP individuals, DSI-healthy individuals and DSI-patients, respectively.

**Conclusion:**

Atlas-labeled ML is comparable to high angular resolution tractography in healthy or hippocampal sclerosis patients. Therefore, rapid identification of the ML location with a single modality of T1 is practical. This protocol would facilitate functional studies and visual field protection during neurosurgery.

## Background

As temporal lobe epilepsies (TLEs) are the most common type of partial seizures, TLE surgeries account for 2/3 of total epilepsy surgeries [1]. Among TLE surgeries, anterior temporal lobectomy (ATL) is the most frequently performed surgery that can help more than 50% of patients become seizure free and achieve an 80% reduction in seizures in more than 70% of cases [2, 3]. Although ATL is widely accepted as a classic and effective surgery for TLE treatment, symptoms of visual field defects (VFDs) are frequently reported among those undergoing ATL treatment, with an incidence ranging from 28 to 52%. Most VFDs are contralateral superior quadrantanopia [4–6]. Damage to the anterior portion of the optic radiation (OR) or Meyer’s Loop (ML) represents the proposed cause of VFD after ATL [7].

Previous studies have visualized ML using diffusion tensor imaging (DTI) to protect visual function [8–14]. However, as it decodes only the average trend of orientation for each voxel, it is challenging for DTI to decode direction from voxels that contain angular fibers (such as sharp angles between the optic tract (OT) and ML and the dramatic anterior extended curvature of fibers in the loop), fanning fibers (such as fanning fibers originating from the lateral geniculate nucleus (LGN)) and crossing fibers [15]; thus, it cannot decode the anatomical features of the ML. Therefore, a low estimation of the anterior extension of ML might exist. This point was supported by previous studies in which the distance from the anterior tip of the temporal lobe to the anterior ridge of Meyer’s loop (dTM) was measured. Although the dTMs were reportedly less than 30 mm in most cadaver studies [16–18], dTMs larger than 37 mm were frequently reported in DTI studies [8, 12]. Thus, different approaches are still needed to facilitate the evaluation of the anterior extension of ML.

Recently developed high angular resolution protocols, such as diffusion spectrum imaging (DSI) or high angular resolution diffusion imaging (HARDI), might be appropriate approaches. These protocols can acquire enormous diffusion directions within a voxel in living humans [15] and decode multiple diffusion trends within a single voxel by accounting for multiple quantitative anisotropy (QA) measures under different b-values [19]. These approaches can improve the quality of visualization of the aforementioned complex anatomical features of ML [15]. In addition to the high angular resolution diffusion protocols, Chamberland et al. [14] used a high magnetic gradient of 300 mT/m and 180 diffusion directions and found that a larger magnetic gradient could facilitate the visualization of the anterior portion of ML. Nevertheless, questions regarding the clinical use of these novel approaches still exist. First, the scanning process of high angular resolution protocols still needs a longer duration. Second, scanners with such a high magnetic gradient may currently be available only in the laboratory.

With these advanced diffusion techniques, in the current study, we presented an OR atlas (ORA) in the averaged Montreal Neurological Institute (MNI) space in which the sprawl (including the anterior extension) of the OR was extracted from multiple groups of the 300 mT/m HARDI data (512 directions), and the anatomical features of the ML atlas were evaluated. Then, we further provided an inverse normalization protocol that could enable the labeling of the atlas in the individual space based on T1 data only (3–7 min). The accuracy of the ORA labeling results was quantitatively evaluated among healthy individuals and patients with hippocampal sclerosis (HS). In addition, we included the atlas, the script for inverse normalization and codes for compatibility of a neuronavigation/robot system.

## Methods

**Fig. 1:**
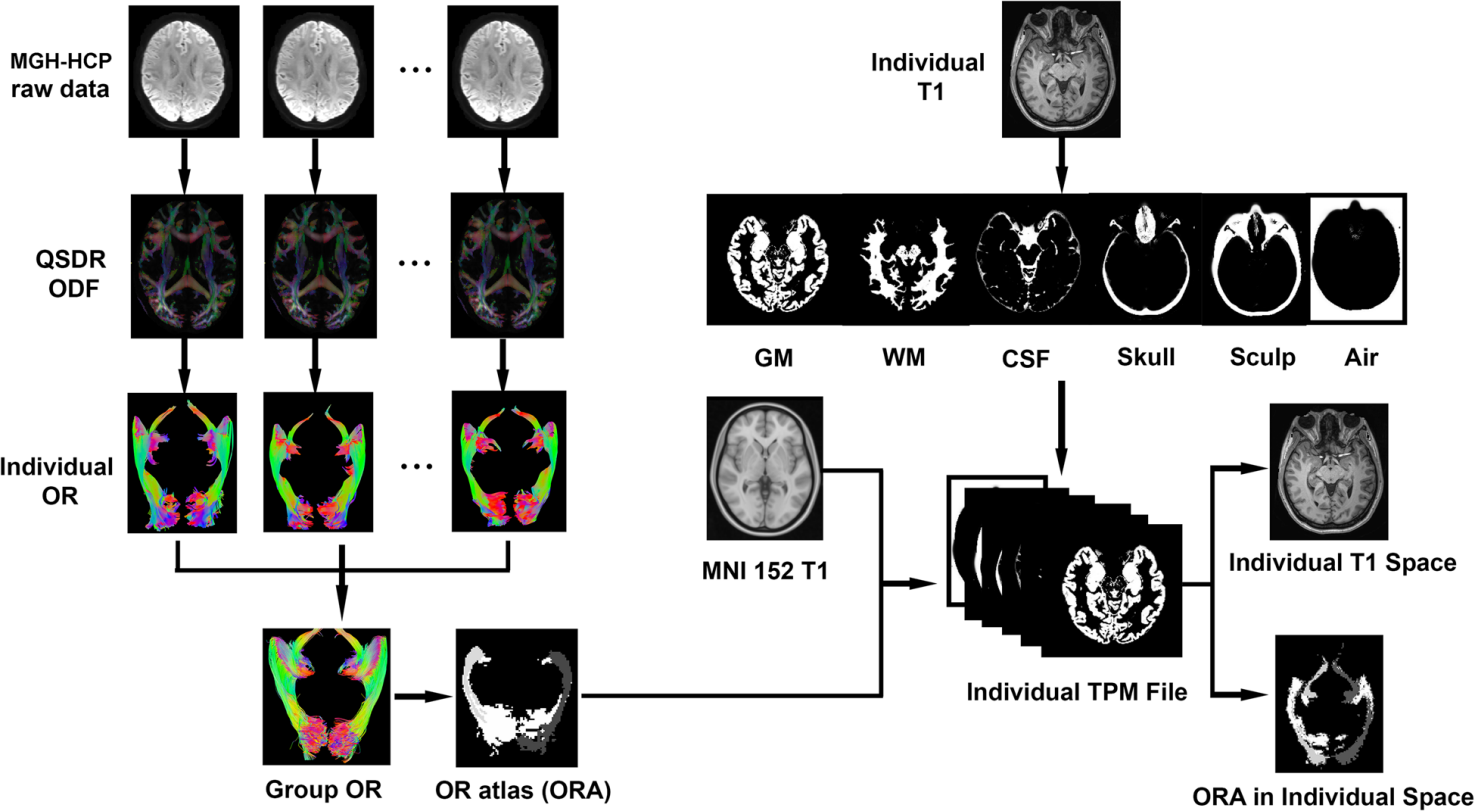
Flow chart demonstrating the process of creating the ORA and labeling the ORA to individual T1 images. Fiber tracking processes were first performed in the 30 QSDR spaces. The resulting fibers were projected together to the same space and converted to regions. The regions were further coregistered to the MNI space; thus, the ORA was created. In the latter procedures, reverse normalization was realized by warping the MNI space to the individual T1 space based on the individual TPM file generated in a previous segmentation procedure

We extracted the common distribution of the OR from 30 samples of Massachusetts General Hospital-Human Connectome Project (MGH-HCP) diffusion data, a group of high-quality datasets that could be acquired only under laboratory conditions, and projected the common distribution of the OR in MNI152 space to create the ORA. The anatomical features of the OR were quantified, and we then tested whether labeling the ORA based on the individual T1 space is reliable by comparing the actual fiber distribution of fiber tracking with the ORA-labeled area in the individual T1 space. This validation was performed for the 30 MGH-HCP cases, eight healthy adults (6 men; 2 women; average age: 24.43 years; age range: 23–27 years) and six consecutive patients (5 men; 1 woman; average age: 25.8 years; age range: 14–48 years) with HS who received a half q-space DSI scanning protocol. Figure 1 summarizes the workflow of the present study. This study was approved by the ethical committee of Xuanwu Hospital.

### Participants and data acquisition

#### The MGH-HCP dataset

Thirty participants from the MGH-HCP (https://db.humanconnectome.org) were involved in the present study. Although the database contains a total of 35 participants, five participants were excluded for compatibility reasons or because their data had obviously low average QA values). These datasets were acquired at MGH with a 3 T scanner, which has a gradient strength of 300 mT/m [20, 21]; to the best of our knowledge, this magnetic gradient is the largest among the released data worldwide. The quality of the diffusion images was improved by the large magnetic gradient, which facilitated the decoding of the fanning shapes and sharp angles within ML. The parameters of the MGH-HCP datasets were as follows: TR: 8800 ms, TE: 57 ms, FOV: 210 mm × 210 mm, and voxel size: 1.5 mm × 1.5 mm × 1.5 mm. The following five shells of b-values were used: 1000 s/mm^2^, 3000 s/mm^2^, 5000 s/mm^2^, 10,000 s/mm^2^ and 10,000 s/mm^2^, with diffusion directions of 64 directions, 64 directions, 128 directions, 128 directions and 128 directions, respectively, for each of these shells (for details of the parameters, please visit: http://protocols.humanconnectome.org/HCP/MGH/). These data were used to create the ORA in an averaged space and validate the usability of this common ORA by projecting the ORA to the T1 space of the 30 MGH-HCP participants.

#### Local DSI dataset

Half q-space DSI data acquisition (258 diffusion directions) was performed with a GE premier MRI scanner (General Electric Healthcare, Waukesha, WI, USA). A 48-channel head coil was used. Head motion was reduced by a head stabilizer. The scanning duration of the DSI protocol was 26 min. The b-values ranged from 0 to 7000 s/mm2. Other parameters were as follows: repetition time (TR): 5548 ms, echo time (TE): 84.1 ms, voxel size: 2 mm × 2 mm × 2 mm, and hyperband acceleration factor: 2. These local DSI datasets were also used to compare the ORA-labeled area and tract distribution in the individual space.

### Creating the ORA in Montreal neurological institute (MNI) space

The creation of the ORA was performed with the 30 MGH-HCP datasets. All the processing of the diffusion images was performed with DSI-studio (http://dsi-studio.labsolver.org) software. We first extracted the orientation distribution within each voxel or the orientation distribution functions (ODFs) to the same space with the q-space diffeomorphic reconstruction (QSDR) algorithm to make the spatial coordination of the tracts comparable across participants [22]. The parameters for this process were as follows: diffusion sampling length ratio: 1.2 and normalization algorithm: constrained diffeomorphic mapping. After this procedure, 30 representations of the ODF map in the QSDR space were acquired. These maps were used for further fiber tracking.

#### Defining the tracking regions

This process was performed with an ODF map in QSDR space. According to the anatomical features of origin and termination, three automatically defined tracking regions were involved in the present study: the LGN, which is the anatomical origin of the OR [17], and the V1 cuneus portion (V1c) or the V1 lingual gyrus portion (V1 l), which is the anatomical termination of the upper or lower division of the OR. Technically, the LGN was defined by the Talairach atlas, while V1c/V1 l was defined by the intersection of the V1 (HCP-MMP atlas) and the cuneus (FreeSurfer DKT atlas)/lingual gyrus (FreeSurfer DKT atlas). These tracking regions were imported from these atlases into the QSDR space through nonlinear registration. Specifically, the LGN was set as the seed, while V1c/V1 l was defined as the region of interest (ROI). Here, the LGN-V1c combination was used to visualize the upper division of the OR (ORu), whereas the LGN-V1 l combination was used to track the lower division of the OR (ORl).

#### Fiber tracking parameters

The QA threshold was set at a value in which most of the orientation signal was distributed within the brain tissue, with as little of the orientation signal falling into the subarachnoid space or ventricles as possible, through the observation of the ODF map. In addition to the threshold, the following tracking parameters were used: angular threshold = 90, step size = 0.5, smoothing = 0.8, min length = 30.0 mm, max length = 300.0 mm, trilinear interpolation and streamlined algorithm. The tracking process was set to terminate if the seed number was 50,000. After this procedure, we obtained 30 groups of OR courses within the QSDR space.

#### Creation of the OR atlas

Despite using high angular resolution data, potential underestimation of the fibers in ML might still exist at the individual level. To overcome this shortcoming, we merged the resulting 30 groups of fibers from the QSDR space of different individuals into a single QSDR space.

The areas in which these fibers were distributed were transformed into “regions”. Thus, we acquired the distribution of four subdivisions across all 30 participants, corresponding to the left ORu, right ORu, left ORl and right ORl specifically. Values of ones, twos, threes and fours were interpolated to the range of these subdivisions accordingly. As this OR distribution was in the QSDR space, it was transferred to MNI space by rigid registration using the FLIRT tool in FSL software (https://fsl.fmrib.ox.ac.uk/fsl/fslwiki/). Thus, we acquired the ORA with high angular resolution data.

### Validating the autolabeling in individual space

As the goal of creating the ORA is mainly to determine the range of OR in the individual T1 space, we compared the range labeled by the ORA in the individual T1 space to the actual fiber tracking results. This process was performed on 30 HCP-MGH participants, eight healthy participants and six HS patients.

#### Warping the atlas to individual space according to T1 images only

The ORA was warped to the individual T1 space with SPM 12.0 software (https://www.fil.ion.ucl.ac.uk/spm/software/download/) and MATLAB (MathWorks, Inc., Natick, MA). Inverse normalization was performed (Fig. 1). Segmentation was first performed on T1 data to create tissue probability maps (TPMs) in individual space. This TPM was adopted as the template, and T1 in the MNI 152 atlas (http://www.bic.mni.mcgill.ca/ServicesAtlases/ICBM152-NLin2009) was used to estimate the space to be warped. During the process, the ORA was specified as the images to be written from the MNI 152 space to the individual T1 space. After the spatial warping, a coregistration process was further performed to the warped ORA to ensure that it was completely aligned to the individual T1 space. The batch script of this warping process has been provided in the supplementary information.

#### Fiber tracking in individual space

Instead of the averaged QSDR space, we reconstructed the ODF map in the individual diffusion space during this process; depending on which process we performed, the fiber tracking with the same tracking method was adapted from the previous steps. Then, the resulting tracts were transferred into a tract density image (TDI) in which the value of each voxel equals the number of tracts passed through the voxel. The TDI was projected to the local T1 space.

#### Accuracy outcome

As the middle 1/3 of the OR has a relatively simple anatomical feature, it would be relatively easy to visualize across different diffusion scanning protocols. Thus, we adopted this portion to validate the accuracy of the reverse normalization by determining the percentage of voxels that had the largest tract density that fell within the labeled range in individual T1 space (codes are presented in supplementary material) or the correspondence rate (CR).

The CR was adopted as the index to estimate the effectivity of the sample size among the local healthy adults and patients first. It was presumed that as long as the areas that had the largest tract density of DSI tracking results overlapped with the ORA-labeled areas, the distances of the ORA and the DSI tracts would be sufficiently adjacent. This deviation between the ORA and the DSI tracts would be tolerable for surgery [23]. Thus, we estimated the CR when the tracts touched the lateral border of the ORA-labeled areas (tolerable CR). This procedure was done by introducing different errors imitating registration deviations in the left and right direction through cyber intervals (a procedure used to detect the transition of intersection and nonintersection of the two regions). Mean and standard deviation across the local healthy adults and patients of the tested CR and the tolerable CR were evaluated according to pre-exams. The mean of the tested CR was evaluated as 75%, the mean of the tolerable CR was 4%, which resulted from cyber intervals, and the standard deviation of the tested CR was 10%. According to the “one sample mean” method (PASS 11.0 software, NCSS, LLC. Kaysville, Utah, USA), with a confidence interval of 95% and power of 0.90, the sample size would be effective as long as it was larger than three cases in each of the groups.

During the experimental procedure, the evaluation of CR was performed on 60 sides of the 30 samples of MGH-HCP diffusion data first. Then, a bootstrap test was adopted by replacing CR samples in 60 sides 10,000 times to verify whether the CR is representative or random in the population of young adults (95% confidence). CR was also observed in the eight healthy adults and six patients with HS to determine the accuracy of the atlas together with the protocol among the local data. For situations in which the CR was obviously lower than the average level in the patients, we further evaluated whether the voxels with maximum tract density fell within the potential surgical safety zone around the ORA-labeled areas (the reported safety distance of the diffusion tractography to the resection zone was 8.7 mm [23]; here, we defined the potential surgical safety zone as 5 mm away from the ORA-labeled area with a more strict criterion).

#### Changing the format for navigation/robot compatibility

With the aim of labeling the ORA in the individual physiological surgical space, we provided a MATLAB script in which the readers could convert the individual T1 images with ORA labeling into a navigation/robot compatible format (Digital Imaging and Communications in Medicine, DICOM).

## Results

### OR distribution in the averaged space

By tracking the ORs in the QSDR space and projecting them together, we obtained fibers illustrating the distribution of the OR across the 30 MGH-HCP participants. Anatomical features, such as sharp angles between the OTs and ML and the fanning shape of ML after originating from the LGN, were adequately presented.

**Fig. 2:**
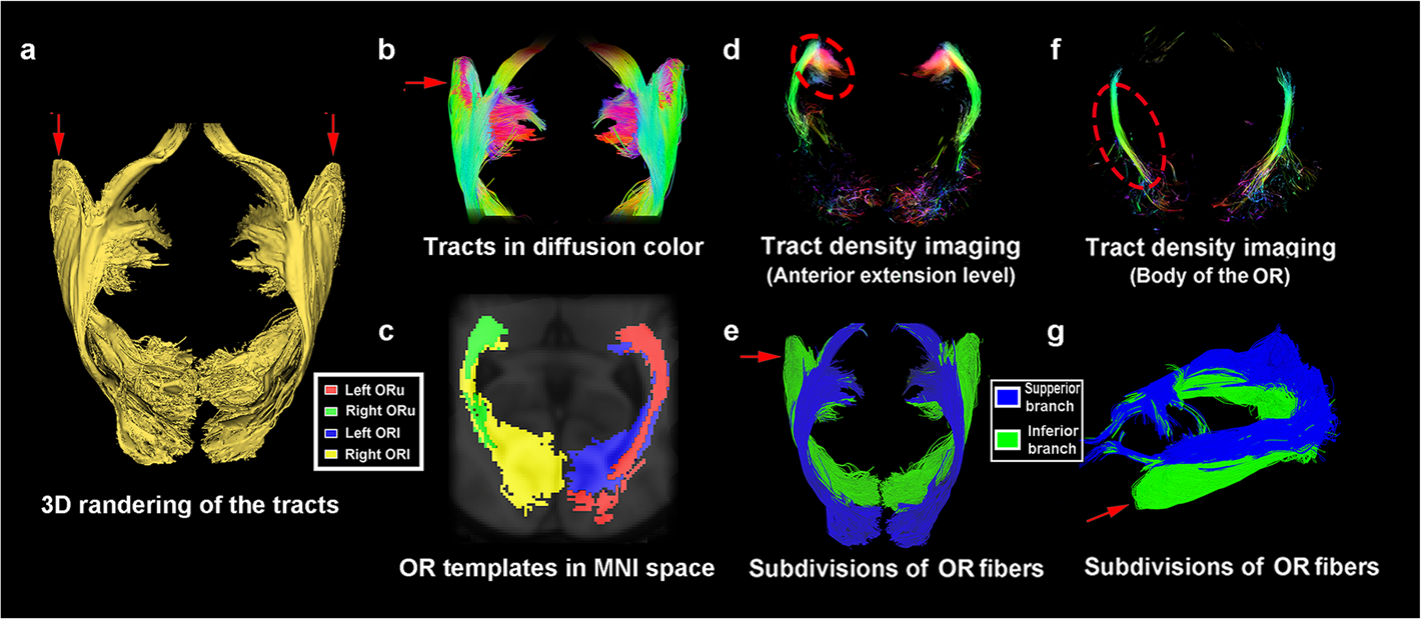
Total OR fibers of 30 MGH-HCP data samples. **a** 3D rendering of the merged OR extracted from 30 sets of data from the MGH-HCP database. The red arrow indicates the visualized anterior extension of the ML; **b** the ML composed of 30 groups of fibers are shown in diffusion; **c** OR atlas labeled in the MNI space**. d** Tract density image (TDI) through the anterior extension level of the OR**.** Slightly decreased tract density could be observed at the ML (dashed circle); **e** Superior view of the total OR fibers. The ORu and ORl are highlighted in blue and green, and the anterior extension (red arrow) is located mainly within the ORl; **f** TDI through the body of the OR**,** showing a concentrated fiber density. **g** Lateral view of the same structure in e

After origination, the OR was observed to emit fibers with fanning angles (Fig. 2), circumvent the temporal horn of the ventricle and move posteriorly while constituting the inferior 2/3 of the lateral ventricle wall. The ORu and ORl were observed in a twisted arrangement (Fig. 2) in which the ORu started in a medial and superior position to the ORl; however, at the posterior portion (approximately at the posterior boundary of the lateral ventricle) of the OR, while still maintaining a superior position, the ORu became slightly lateral to the ORl.

The distances from the center of the LGN to the most anterior ridge of the ML were 40.7 mm on the left and 41.1 mm on the right side, respectively, while the dTMs were 23.1 mm and 26.41 mm on the left and right, respectively. In the TDI of the OR, which contains 30 groups of tracts, a slightly decreased number of tracts could be observed at the anterior extension of the ML (300–400 tracts per voxel) relative to the body of the OR (400–500 tracts per voxel) (Fig. 2).

### Validation in individual space

**Fig. 3:**
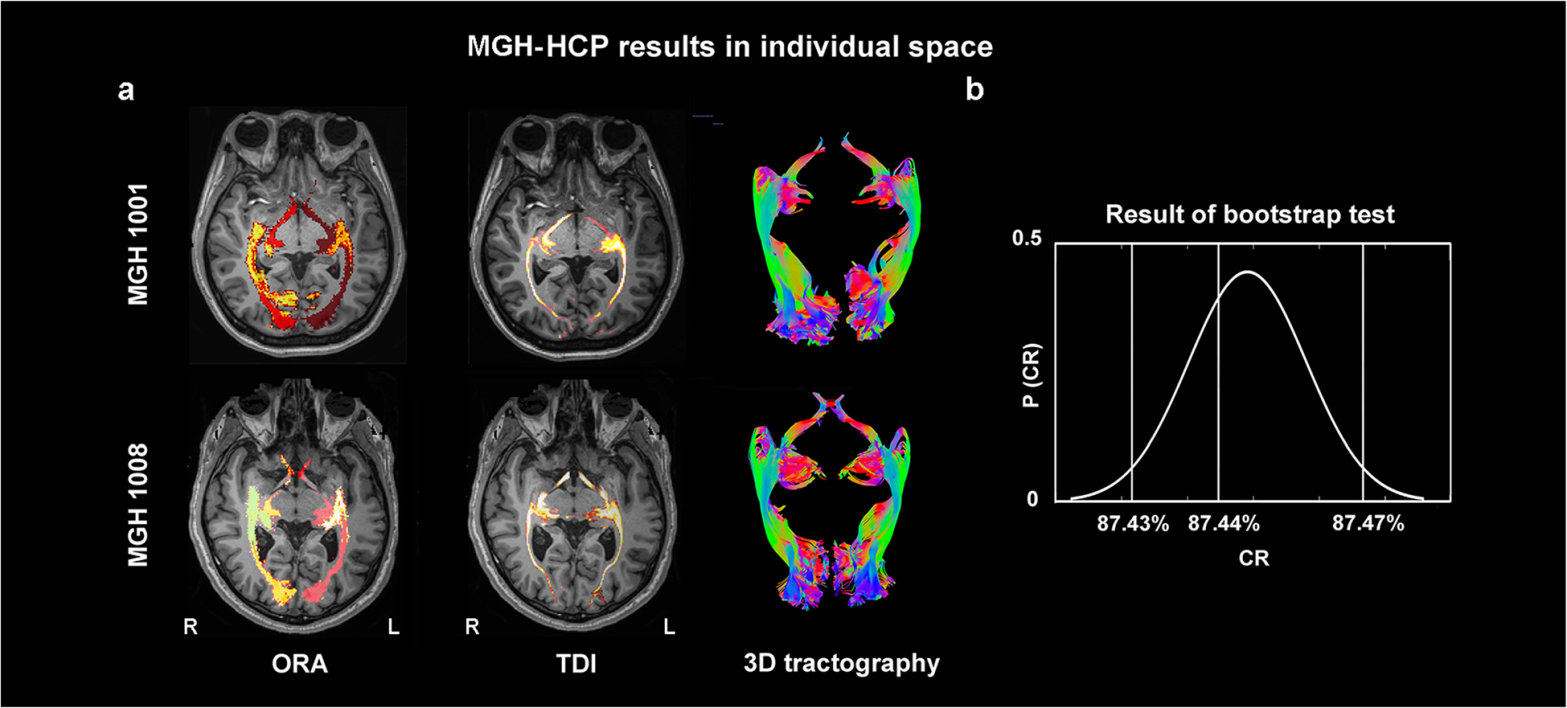
Comparison of the ORA-labeled region and TDI region in the MGH-HCP individual space. **a** Examples of the labeling outcome in participants of round (upper row) and long head-shape (lower row). The distribution of TDI (second column) is overall coincident with the sprawl of the ORA in the participant-specific individual space. 3D tractographies show the fibers that were used to calculate the TDI. **b** Results of the bootstrap test**.** This curve shows that the average CR across the 30 MGH-HCP participants was located within the 95% confidence interval, indicating that the congruence between the TDI and the ORA in the MGH-HCP individuals might be representative at the population level

**Fig. 4:**
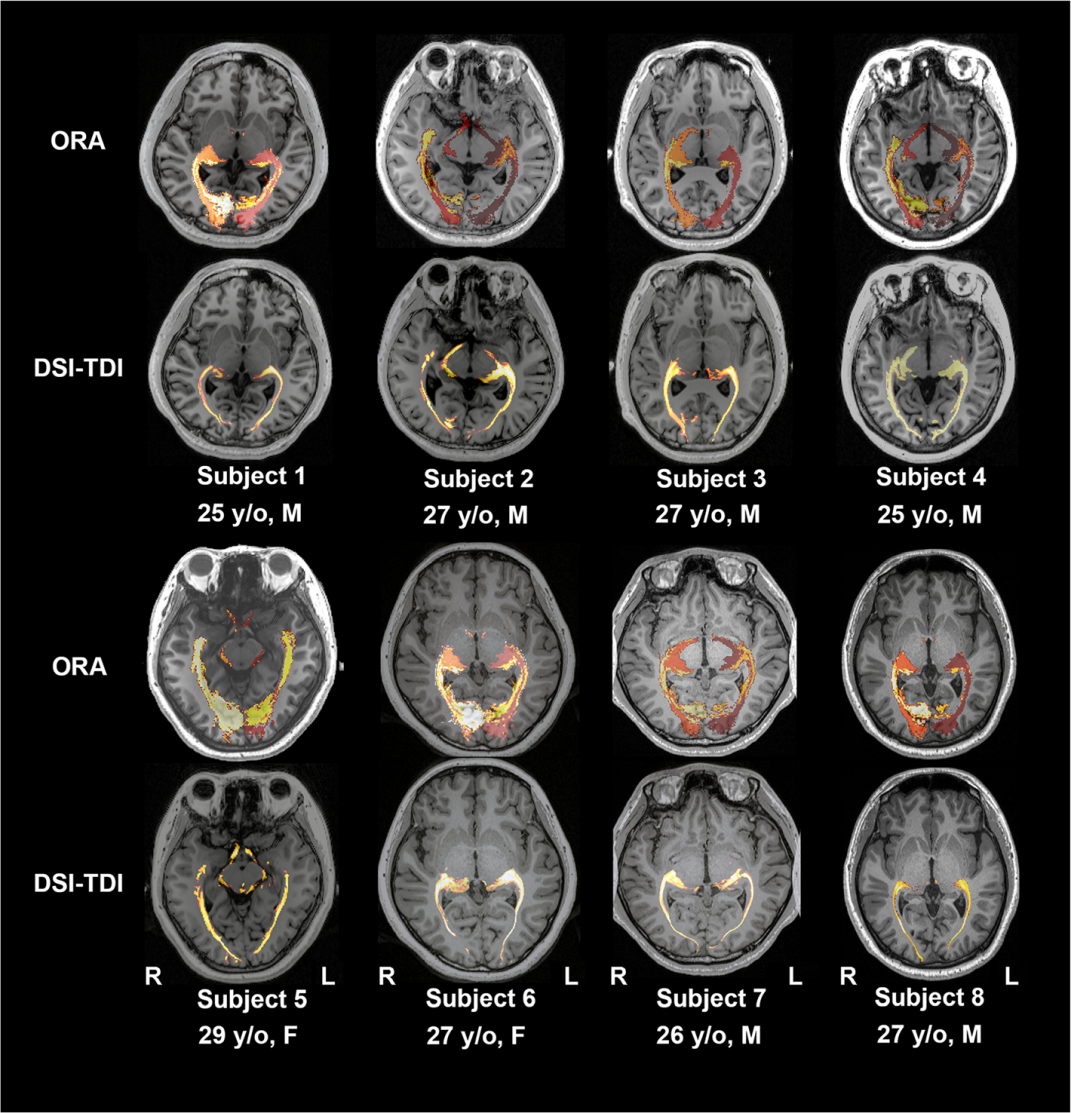
Comparison of the ORA-labeled region and TDI region in eight healthy adults. Rows of odd numbers show the results of the ORA-labeled area, while the rows of even numbers demonstrate the sprawl of fibers in high-definition fiber tractography

**Fig. 5:**
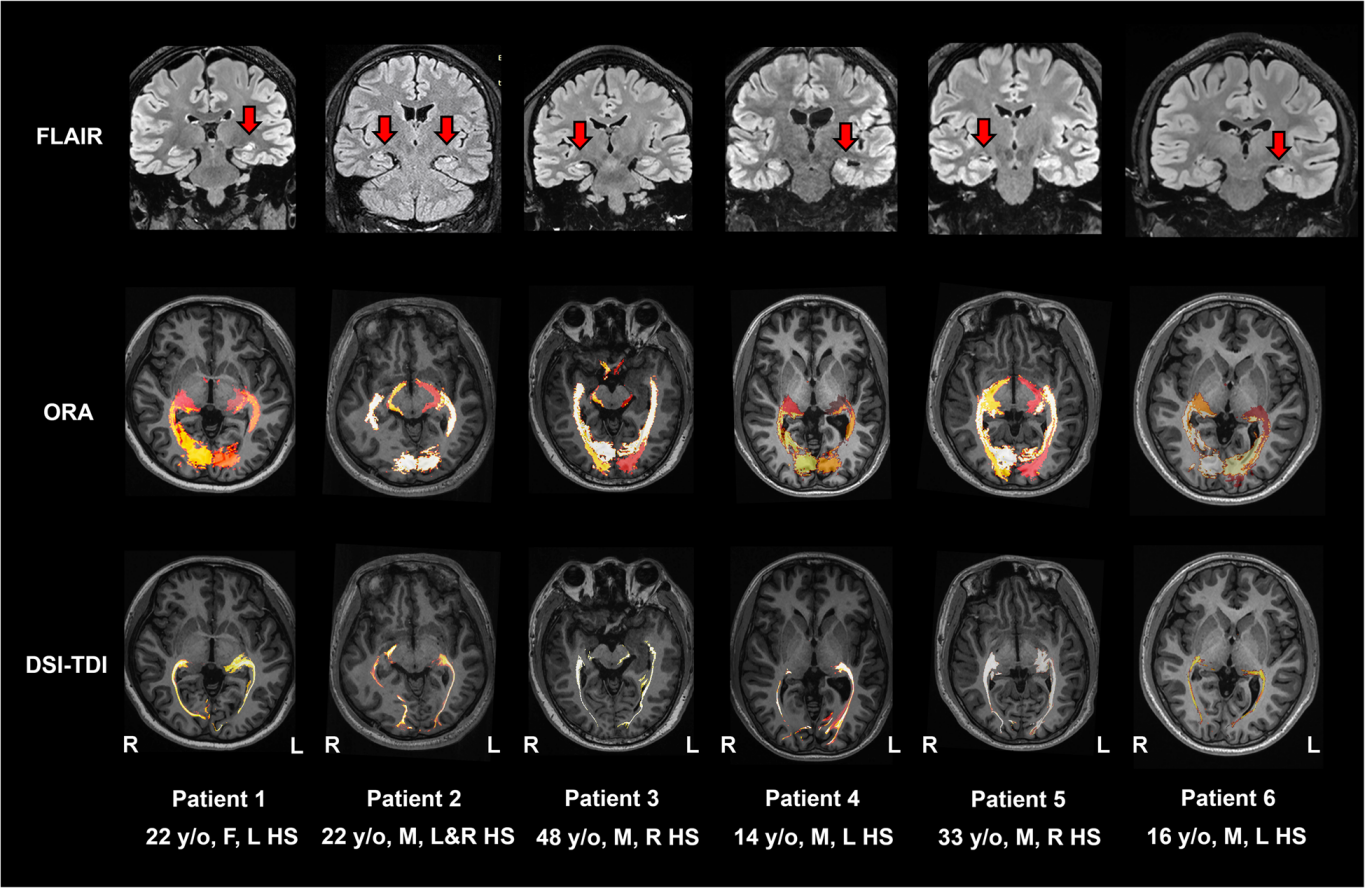
Comparison of the ORA-labeled region and TDI region in six HS patients. **a** Structural findings of the six patients, which manifested as an increased signal together with atrophy of the hippocampus (red arrows); **b** ORA-labeled regions in the individual space; c. TDI distributed regions. Overall, the TDI is located within the range suggested by the ORA

In all 30 MGH-HCP participants (examples are shown in Fig. 3), eight local healthy participants (Fig. 4) and six local HS participants (Fig. 5), the averaged ORA was warped into the individual space and compared with the results of fiber tracking. With the created individual-specific TPM, the ORA successfully labeled a total of 88 sides of the OR based on T1 structural images alone. The sprawl of the ORAs in the individual space was observed to fit well with the curves of the surrounding anatomical structures, such as the ventricle and the shape of the brain (long or short in the anterior-posterior axis).

Concerning the CR, among the 60 sides of the OR in the 30 MGH-HCP datasets (Fig. 3), an average of 87.44% ± 6.92% of the voxels that had the maximal tract density fell within the range of the individualized ORA. A bootstrap test of 10,000 replacements revealed that the CR was within the 95% confidence interval, indicating that the CR sample of the MGH-HCP data was not random (Fig. 3).

We further labeled the ORA to T1 space in eight healthy adults (Fig. 4), and the CR was 80.81 ± 10.62% (a total of 16 sides). Among the six patients with HS (Fig. 5), the CR was 72.83% ± 14.03% among 12 sides of all the patients. Specifically, in two patients with the most severe hippocampal atrophy, the lowest CR values of 49.88% (healthy side of patient 1) and 52.77% (affected side of patient 4) were observed. However, all the voxels with the maximum tract density in fiber tracking still fell within the safety zone indicated by the ORA-labeled area.

With the script we provided, all the results were successfully transferred to the DICOM format and passed the compatibility test using the surgical systems.

## Discussion

In the present study, we created an atlas of the OR with adequate evaluation of the anterior extension of ML using high angular resolution diffusion data acquired with the 300 mT/m connectome scanner of the HCP-MGH consortium. Furthermore, we presented a labeling approach for the sprawl of the OR in the individual space with T1 structural images alone. The accuracy of the labeling was proven to be comparable to the results of DSI or HARDI tractographies in individual space among healthy participants and HS patients.

### Anterior extension of Meyer’s loop

Regarding the anterior extension of ML, in the present study, the dTMs were 23.1 mm and 26.41 mm in the ORA on the left and right sides, respectively. Several previous DTI studies were carried out to explore this distance. Taoka et al. [8] reported a dTM of 36.6 mm (ranging from 30.0 to 43.2 mm), while James et al. [12] reported distances of 37.44 ± 4.7 mm (range: 32.2–46.6 mm) and 39.08 ± 4.9 mm (range: 34.3–49.7 mm) on the left and right sides, respectively, in their DTI studies. In addition to DTI studies, the dTM was measured comprehensively in previous anatomical studies. Ebeling and Reulen [16] reported a dTM of 27 ± 3.5 mm through measurements of 25 hemispheres, and Peuskens et al. [17] presented a dTM of less than 30 mm in their studies. Parrage et al. [18] reported an average dTM of 28.4 mm in their microsurgical anatomical study of 40 brain hemispheres. The dTM seems to be consistently larger in canonical DTI studies than in anatomical studies, which indicates that a low estimation of ML might exist with DTI. The dTM of the ORA is comparable to the previous anatomical literature because of the high magnetic gradient and angular resolution of the HCP-MGH data. That is, the template created in the present study might contain adequate information of the anterior extension of ML by averaging the 30 MGH-HCP datasets together.

We subdivided the ORA into subregions, ORu and ORl, and the twisting sprawl of these subdivisions was consistent with previous anatomical studies [24]. The most anterior extension portion of ML was observed to be mainly constituted by the ORl in the present study. As the ORl terminated at the lingual area where information of the contralateral superior visual field was deposited, the contralateral superior visual quadrium should be the most vulnerable area to injury during ATL. This idea is in accordance with previous experiences [7].

### Accuracy and representativeness of the ORA in individual space

Reverse normalization has been shown to be reliable in robot/neuronavigational systems in recent years [25, 26]. In the present study, a high CR was reached over the ORs of 88 hemispheres in the individual space, which means that in most cases, the voxels with the largest tract density would fall within the labeled range. The bootstrap test specific to the CR was performed in the MGH-HCP subgroup, and the CR was in the 95% confidence interval, indicating that the ORA labeling method is representative rather than random at the population level.

Among the patients, the CR was slightly lower than that in the MGH-HCP subgroup and DSI-healthy subgroup, and severe atrophy of the mesial temporal lobe could result in 1–2 mm dislocation. However, this reduction in the CR is acceptable, as a safety distance of 5–10 mm is typically required in neuronavigation [23]. In addition, as the hippocampus is mainly located posterior to ML [17, 18], in principle, dislocations resulting from the hippocampus would be more pronounced at the posterior portion of ML than at the anterior portion. Therefore, localization of the anterior extension of ML is practical.

### Scanning duration and clinical compatibility

Most of the labeling process of the ORA to the individual space was based entirely on 3D T1 images, which could be acquired within 3–7 min during daily clinical practice. In contrast, the diffusion data acquisition process in the MGH-HCP scanning protocol requires a duration of more than 89 min in the 300 mT/m connectome scanner (http://protocols.humanconnectome.org/HCP/MGH/). Similarly, the DSI protocol used in the present study requires a period of 26 min. All of the HARDI/DSI data in the present study require the utilization of an advanced scanner. Thus, we presented an approach that could adequately evaluate the anterior extension of ML within a short and clinically applicable duration.

### Limitations

Limitations exist in the present study. First, as the reverse normalization could be performed only within non-space occupying brains, the ORA in the present study should not be used for patients whose brain tissue is compressed and severely affected by a lesion. Thus, the aim of the atlas is mainly to serve MTL or other MRI-negative temporal epilepsy surgeries. In addition, in the present study, we mainly compared the ORA-labeled area and the sprawl of OR in high-resolution tractography, while intraoperative evaluation of high-resolution tractography-guided neuronavigation was presented in our previous works [27]. Overall, labeling with the atlas is practical according to the present study.

## Conclusions

In the present study, we created an atlas with 30 high angular resolution and high magnetic gradient diffusion datasets from the MGH-HCP database. The anterior extension and fanning shape as well as the sharp angle of ML were adequately represented in the atlas. In routine 3D T1, which was acquired in a relatively short period, we successfully labeled the ORA with a reverse normalization protocol. The labeled region was highly congruous with high angular resolution tractographies in both healthy participants and HS patients. Therefore, rapid identification of the ML location with a single modality of T1 is practical. This process would facilitate visual field protection in patients, especially those who are uncooperative.

## Data Availability

The MGH-HCP datasets can be downloaded from http://protocols.humanconnectome.org/HCP/MGH/ The local DSI data in this study are available from the corresponding author on request.

## Abbreviations

ALT: Anterior temporal lobectomy
CR: Correspondence rate
DSI: Diffusion spectrum imaging
DTI: Diffusion tensor imaging
dTM: Distance from the anterior tip of the temporal lobe to the anterior ridge of Meyer’s loop
HARDI: High angular resolution diffusion imaging
HS: Hippocampal sclerosis
LGN: Lateral geniculate nucleus
MGH-HCP: Massachusetts General Hospital-Human Connectome Project
ML: Meyer’s loop
MNI: Montreal Neurological Institute
ODF: Orientation distribution functions
OR: Optic radiation
ORA: OR atlas
ORl: Lower branch of optic radiation
ORu: Upper branch of optic radiation
OT: Optic tract
QSDR: Q-space diffeomorphic reconstruction
ROI: Region of interest
TDI: Tract density image
TLE: Temporal lobe epilepsies
TPM: Tissue probability maps
V1 l: V1 lingual gyrus portion
V1c: V1 cuneus portion
VFD: Visual field defects
